# Spatiotemporal analysis of tuberculosis in the Hunan Province, China, 2014–2022

**DOI:** 10.3389/fpubh.2024.1426503

**Published:** 2024-08-08

**Authors:** Guojun Huang, Zuhui Xu, Liqiong Bai, Jianjun Liu, Shicheng Yu, Hongyan Yao

**Affiliations:** ^1^Chinese Center for Disease Control and Prevention, Beijing, China; ^2^Department of Science and Education, Hunan Chest Hospital, Changsha, China; ^3^Department of Tuberculosis Control and Prevention, Hunan Chest Hospital, Changsha, China; ^4^Hunan Chest Hospital, Changsha, China

**Keywords:** tuberculosis, spatiotemporal analysis, spatial autocorrelation analysis, clustering, China

## Abstract

**Background:**

Pulmonary tuberculosis (PTB) is a major infectious disease that threatens human health. China is a high tuberculosis-burden country and the Hunan Province has a high tuberculosis notification rate. However, no comprehensive analysis has been conducted on the spatiotemporal distribution of PTB in the Hunan Province. Therefore, this study investigated the spatiotemporal distribution of PTB in the Hunan Province to enable targeted control policies for tuberculosis.

**Methods:**

We obtained data about cases of PTB in the Hunan Province notified from January 2014 to December 2022 from the China Information System for Disease Control and Prevention. Time-series analysis was conducted to analyze the trends in PTB case notifications. Spatial autocorrelation analysis was conducted to detect the spatial distribution characteristics of PTB at a county level in Hunan Province. Space-time scan analysis was conducted to confirm specific times and locations of PTB clustering.

**Results:**

A total of 472,826 new cases of PTB were notified in the Hunan Province during the 9-year study period. The mean PTB notification rate showed a gradual, fluctuating downward trend over time. The number of PTB notifications per month showed significant seasonal variation, with an annual peak in notifications in January or March, followed by a fluctuating decline after March, reaching a trough in November or December. Moran’s I index of spatial autocorrelation revealed that the notification rate of PTB by county ranged from 0.117 to 0.317 during the study period, indicating spatial clustering. The hotspot areas of PTB were mainly concentrated in the Xiangxi Autonomous Prefecture, Zhangjiajie City, and Hengyang City. The most likely clustering region was identified in the central-southern part of the province, and a secondary clustering region was identified in the northwest part of the province.

**Conclusion:**

This study identified the temporal trend and spatial distribution pattern of tuberculosis in the Hunan Province. PTB clustered mainly in the central-southern and northwestern regions of the province. Disease control programs should focus on strengthening tuberculosis control in these regions.

## 1 Introduction

Pulmonary tuberculosis (PTB) is a major infectious disease that seriously threatens human health. According to the WHO Global Tuberculosis Report 2023 (1), an estimated 10.6 million new cases of PTB occurred globally in 2022, with 1.3 million deaths. PTB remains an important public health problem. After decades of efforts, the incidence of tuberculosis has gradually declined in China. However, the incidence of tuberculosis within the country varies by region, with some areas still experiencing major outbreaks (2, 3). The Hunan Province, located in central-southern China, has reported high numbers of cases and PTB notification rates. In 2022, 43,976 new PTB cases were notified, with a notification rate of 66.40/100,000, which was higher than the national average. Therefore, more effective control strategies are urgently needed to curb the spread of tuberculosis in the Hunan Province.

Spatiotemporal analysis methods have been used extensively to investigate the distribution and variation patterns of PTB in recent years. Studies have been conducted in several countries, including Russia (4), Brazil (5, 6), Uganda (7), and Peru (8). In China, researchers such as Zhang et al. (9) have studied the spatiotemporal distribution characteristics of tuberculosis at a provincial level, whereas others such as Liu et al. (3) have conducted similar analyses at a prefecture level. Studies of the spatiotemporal distribution of tuberculosis have been conducted in Beijing (10), Zhejiang (11, 12), Chongqing (13), and Qinghai (14) Provinces. In the Hunan Province, Alene et al. (15) examined spatiotemporal distribution patterns, and Zheng et al. (16) investigated the spatial clustering and hotspot areas of smear-positive PTB notifications in the Hunan Province in 2012 and 2013. However, to our knowledge, no comprehensive analysis has been conducted on the spatiotemporal distribution of PTB in the whole of the Hunan Province. The current spatiotemporal distribution and trends in the spatiotemporal distribution of PTB in the Hunan Province remain unclear and warrant further investigation.

This study aimed to analyze the temporal distribution characteristics of tuberculosis in the Hunan Province, examining the clustering of PTB notifications at a county level to identify hotspot and cold-spot areas of incidence. Through spatiotemporal scanning analysis, this study aimed to pinpoint specific locations of tuberculosis incidence clustering and assess the magnitude of disease risk in these clusters. By identifying the key regions of PTB incidence in the Hunan Province, these results provide a source of reference for developing targeted tuberculosis control strategies.

## 2 Materials and methods

### 2.1 Overview of the study area

**Figure 1:**
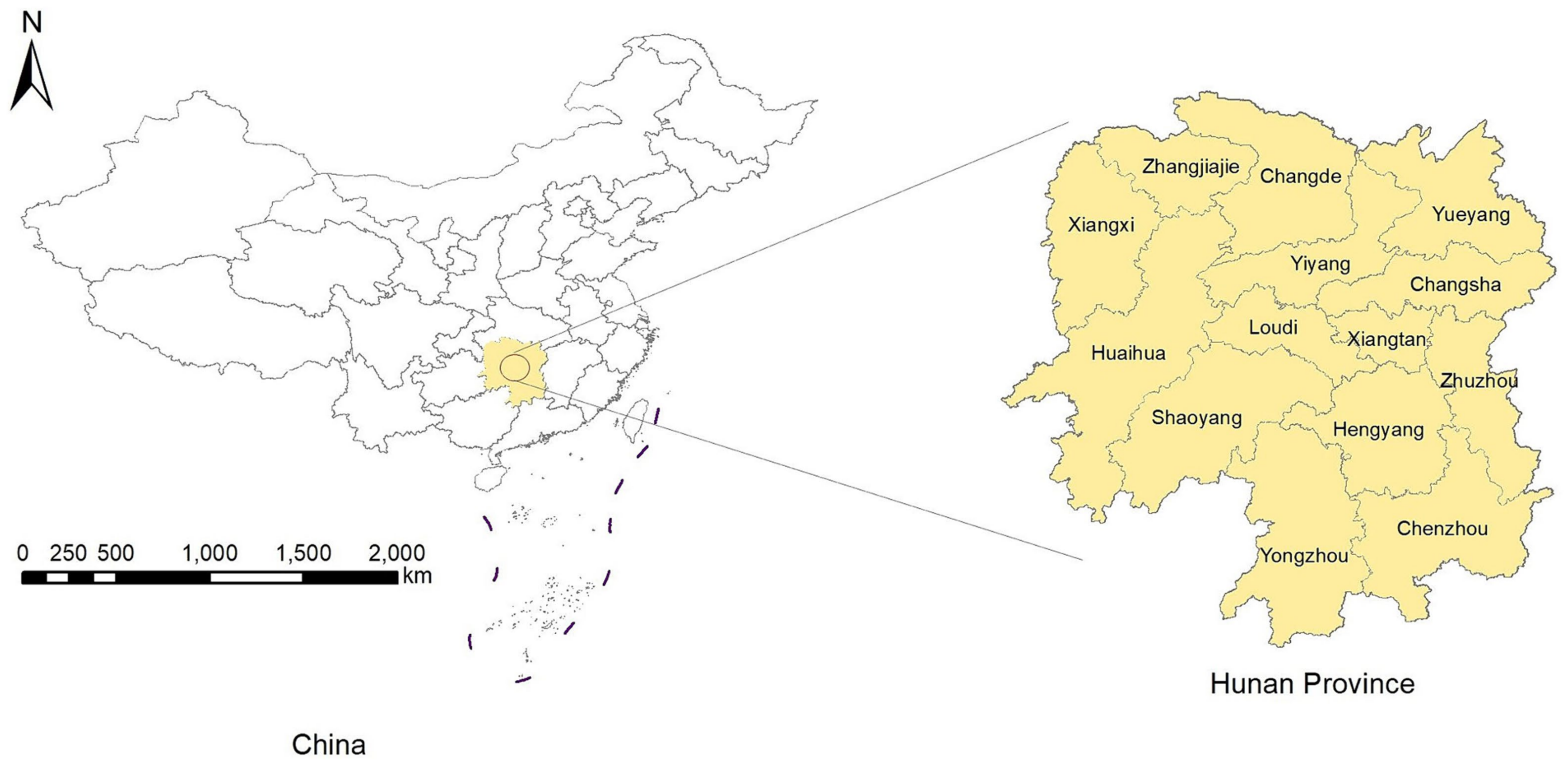
Location of the Hunan Province.

The Hunan Province is in the central-southern region of China (Figure 1), which covers an area of 211,800 square kilometers. Its terrain is characterized by mountains and hills, with mountainous areas covering 51.2%, hills and plateaus covering 29.3%, plains covering 13.1%, and water surfaces covering 6.4% of the total provincial area. The number of permanent residents was 66.04 million in 2022. The Hunan Province includes 14 prefecture-level administrative divisions, with 122 county-level administrative divisions (17).

### 2.2 Data collection

Data on PTB notifications for each county in the Hunan Province from January 2014 to December 2022 were download from the China Information System for Disease Control and Prevention (CISDCP). The dataset included the number of PTB notifications for each county by month. PTB notifications included both laboratory-confirmed and clinically diagnosed cases. From January 2014 to April 2018, the diagnostic criteria for tuberculosis were based on the National Health Commission of the People’s Republic of China WS 288-2008, and from May 2018 to December 2022, the diagnostic criteria for tuberculosis were based on the National Health Commission of the People’s Republic of China WS 288-2017 (18). Population data for each county in the Hunan Province from 2014 to 2022 were obtained from the Hunan Statistical Yearbook (2015–2023). To calculate the mean PTB notification rate, we summed the number of cases notified by county for the 9-year period and divided them by the total population of each county for the same period.

The overall PTB notification rate and the notification rate of laboratory-confirmed cases of PTB were used as indicators of the effectiveness of control programs. The overall PTB notification rate included laboratory-confirmed cases, laboratory-negative cases, and cases with no laboratory results. According to the 2019 notification on the adjustment of the classification of infectious disease reports for PTB (19), laboratory-confirmed cases of PTB include smear-positive cases; smear-negative, culture-positive cases; and cases testing positive on nucleic acid amplification tests (NAATs). Since January 1, 2017, cases of rifampicin-resistant tuberculosis have been recorded in CISDCP (20). Laboratory-negative PTB refers to patients with PTB whose sputum smears and cultures are both negative, and NAAT results are negative or unavailable. PTB with no laboratory results refers to patients with tuberculosis symptoms or tuberculous pleurisy, but no sputum smear, culture, or NAAT results.

### 2.3 Statistical analysis

#### 2.3.1 Time-series analysis

In the time-series analysis, we analyzed the number of notified PTB cases in the Hunan Province from January 2014 to December 2022 (108 months). Using time as the horizontal axis and the number of notified PTB cases per month as the vertical axis, we plotted a curve showing the monthly PTB cases in the Hunan Province over time. The tuberculosis incidence was analyzed by observing the trend in the curve. The analyses were conducted using Microsoft Excel 2019 (Microsoft Corporation, Redmond, WA, United States).

#### 2.3.2 Spatial autocorrelation analysis

Spatial autocorrelation analysis was conducted to investigate whether the observed values at a particular location in a spatial area were correlated with similar observed values in neighboring areas. The analysis included global and local spatial autocorrelation. Global spatial autocorrelation can be classified into positive spatial autocorrelation, negative spatial autocorrelation, and spatial randomness (i.e., random distribution) based on the spatial distribution characteristics. Spatial autocorrelation analysis quantifies the type of spatial data correlation, explores clustering, and examines the process through which spatial features change over time, thereby identifying risk factors for disease in the study region. For the global spatial autocorrelation analysis, we used Moran’s I (21), which is widely applied as a statistical index in spatial epidemiology (11, 13). The Moran’s I index can range between −1 and 1. A positive Moran’s I value closer to 1 indicates higher clustering, whereas a negative value closer to −1 suggests a more dispersed distribution. A value of zero indicates a random distribution. We used this method to confirm the spatial distribution characteristics of the PTB notification rate at a county level in Hunan Province from 2014 to 2022. The Z-score was calculated to assess the significance of the Moran’s I estimate. If Moran’s I is greater than 0 and the Z-score is greater than or equal to 1.96, the distribution of disease is assumed to be spatially clustered and statistically aggregated (22).

Local spatial autocorrelation analysis is used to describe spatial correlation patterns, locate specific clustering areas, and investigate local spatial instability, thus revealing spatial heterogeneity among data. In this study, the local Gi^*^ statistic was calculated to assess local spatial autocorrelation (23) and to identify hotspot and cold-spot areas of PTB notifications. A significant positive value, such as Gi* ≥ 1.96, indicates that high values in the locality are clustered more than those in other areas. Conversely, a significant negative value, such as Gi* ≤ −1.96, indicates that low values in the locality are clustered less than those in other areas. These analyses were conducted using ArcGIS 10.2 software (ESRI Inc., Redlands, CA, United States).

#### 2.3.3 Space-time scan statistic

The space-time scan statistic was proposed by Kulldorff (24) in 1995 to observe and infer the spatiotemporal clustering of diseases. It can detect abnormal changes in the number of occurrences of a specific event (disease) within a spatiotemporal range and test whether these changes are due to random variation. That is, it investigates whether disease clustering exists within the study region, the exact location of the clustering, and the magnitude of clustering risk, and tests whether this clustering has statistical significance. Currently, it is widely used in tuberculosis research (3, 11, 13).

In this study, the space-time scan statistic model used a Poisson model and focused on areas with high PTB notification rates. The space-time scan window is a cylinder in which both spatial and temporal dimensions are constantly changing, corresponding to changes in the radius and height of the cylinder base. Simultaneously, the center of the circle at the base of the cylinder moves between the center points of each spatial unit. Each time the center, radius, and height of the cylindrical window change, a log-likelihood ratio (LLR) is calculated to compare the risk inside and outside the window. The space-time scan uses the Poisson model for every position and scale of the space-time scan window. The null hypothesis assumes that the spatial distribution of the disease is completely random, whereas the alternative hypothesis suggests an increased risk of disease occurrence inside the scan window relative to outside the scan window. For each scan window, the LLR test statistic is calculated based on the disease occurrences inside and outside the window to compare the risk. The window with the maximum LLR is considered the most likely cluster, known as the primary cluster, whereas other windows displaying statistically significant LLR values are defined as secondary clusters (25). These analyses were conducted using SaTScan version 9.5 (Kulldorff, Boston, MA, United States).

### 2.4 Ethical review

This study was approved by the Medical Ethics Committee of Hunan Chest Hospital. All personal information and privacy protection were carried out in accordance with the ethical requirements. The requirement for informed consent did not apply because the notification data were aggregated, and no individual-level data were analyzed.

## 3 Results

### 3.1 Overview of PTB in the Hunan Province

**Figure 2:**
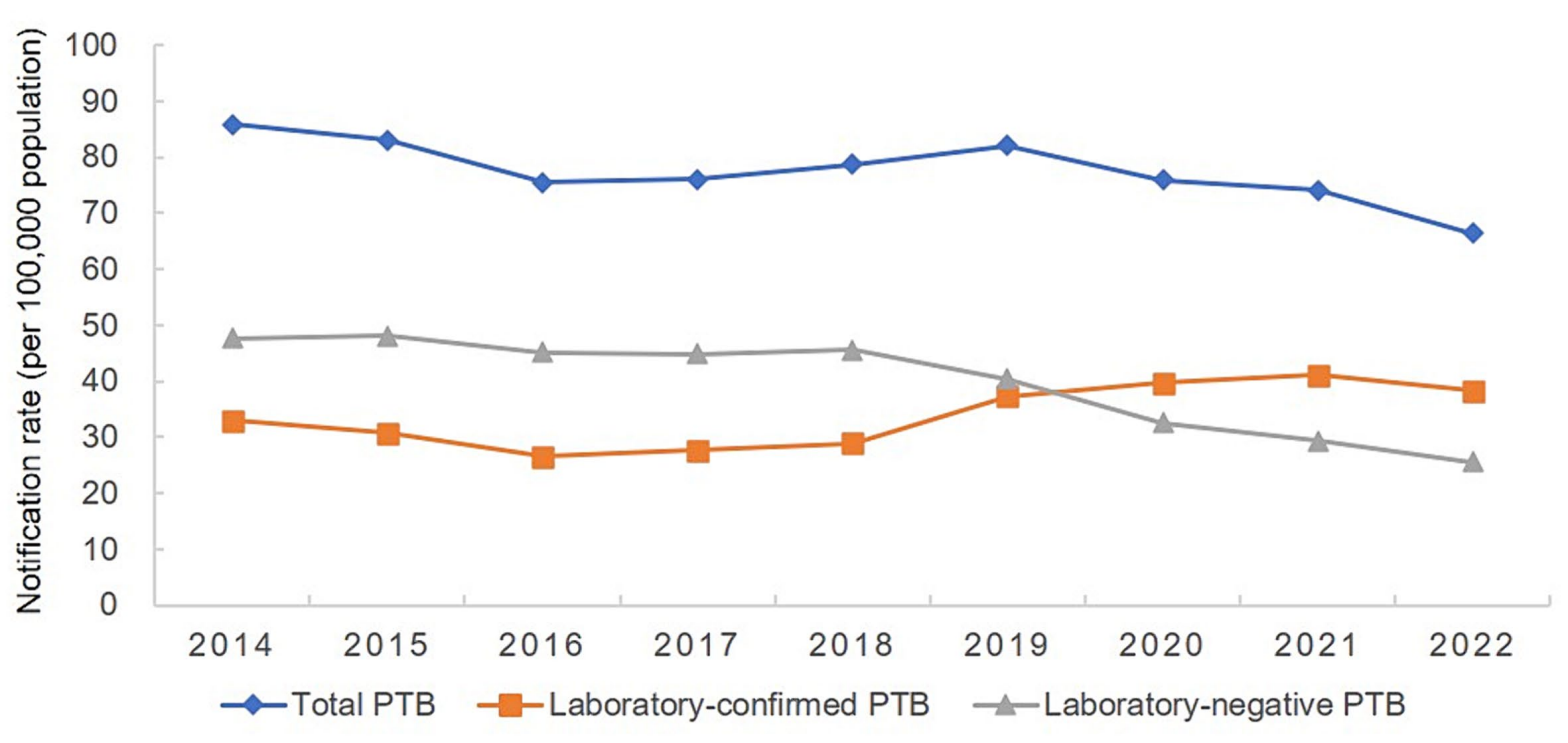
Annual pulmonary tuberculosis notification rate in the Hunan Province, China from 2014 to 2022.

From 2014 to 2022, 472,826 cases of PTB were notified in the Hunan Province; of them, 205,176 cases were laboratory-confirmed, 243,571 cases were laboratory-negative, and 24,079 cases did not have information available on microbiological status. The percentage of laboratory-confirmed PTB increased from 38.43% in 2014 to 57.74% in 2022, whereas the percentage of cases with negative results decreased from 55.49% in 2014 to 38.56% in 2022 (Table 1). The mean annual PTB notification rate in the Hunan Province between 2014 and 2022 was 77.55/100,000. The mean annual PTB notification rate in the province decreased from 85.88/100,000 in 2014 to 66.40/100,000 in 2022, showing a gradual downward trend with fluctuations. The annual notification rate of laboratory-confirmed PTB increased from 33.00/100,000 in 2014 to 38.34/100,000 in 2022, showing a fluctuating gradual upward trend (Figure 2). In contrast, the notification rate of laboratory-negative PTB decreased from 47.65/100,000 in 2014 to 25.60/100,000 in 2022, showing a downward trend (Figure 2).

**Table 1:** Laboratory confirmation of cases of PTB notified in the Hunan Province, China from 2014 to 2022.

Year	Laboratory-confirmed^a^ *n* (%)	Laboratory-negative *n* (%)	No laboratory results available *n* (%)	Total number of cases of PTB
2014	22,080 (38.43)	31,884 (55.49)	3,494 (6.08)	57,458
2015	20,653 (36.93)	32,398 (57.94)	2,868 (5.13)	55,919
2016	17,962 (35.11)	30,552 (59.72)	2,643 (5.17)	51,157
2017	18,848 (36.32)	30,632 (59.03)	2,413 (4.65)	51,893
2018	19,828 (36.70)	31,284 (57.91)	2,911 (5.39)	54,023
2019	25,740 (45.46)	27,893 (49.26)	2,987 (5.28)	56,620
2020	27,433 (52.21)	22,501 (42.83)	2,605 (4.96)	52,539
2021	27,245 (55.32)	19,475 (39.54)	2,530 (5.14)	49,250
2022	25,387 (57.74)	16,952 (38.56)	1,628 (3.70)	43,967
Total	205,176 (43.39)	243,571 (51.52)	24,079 (5.09)	472,826

a Laboratory-confirmed cases of PTB include smear-positive cases; smear-negative, culture-positive cases; and cases diagnosed based on a positive nucleic acid amplification test (e.g., Xpert MTB/RIF).

PTB, pulmonary tuberculosis.

### 3.2 Temporal trends in PTB case notifications

**Figure 3:**
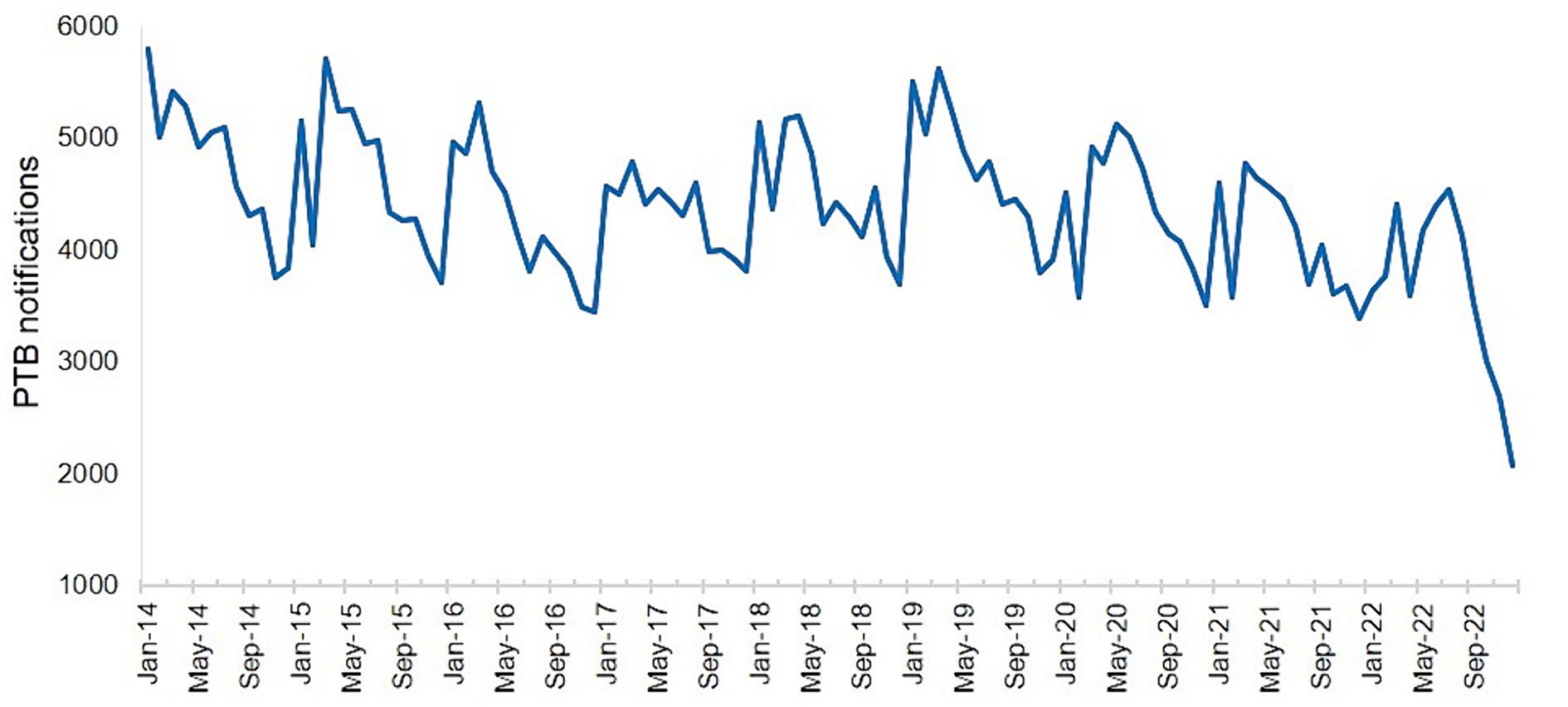
Number of notified cases of pulmonary tuberculosis in the Hunan Province by month from January 2014 to December 2022.

The number of PTB notifications per month showed significant seasonal variation from 2014 to 2019 (Figure 3). The number of notifications peaked in January or March every year, followed by a fluctuating decline after March, and reached a trough in November or December. However, this pattern was disrupted from 2020 to 2022, with peaks occurring in June, April, and July, in 2020, 2021, and 2022, respectively, although the troughs still occurred in December each year.

### 3.3 Spatial distribution of PTB case notifications

**Figure 4:**
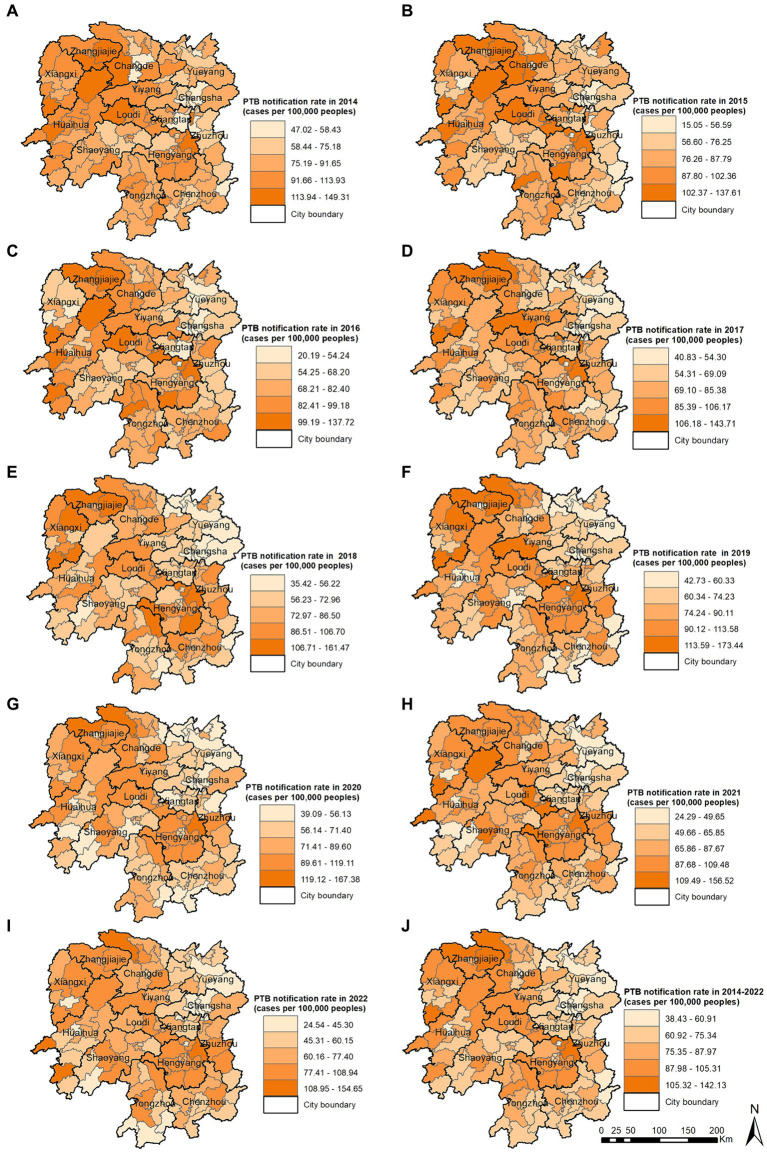
Pulmonary tuberculosis notification rate in 2014 **(A)**, 2015 **(B)**, 2016 **(C)**, 2017 **(D)**, 2018 **(E)**, 2019 **(F)**, 2020 **(G)**, 2021 **(H)**, 2022 **(I)**, 2014–2022 **(J)**.

The PTB notification rate in the Hunan Province between 2014 and 2022 varied by county and district (Figure 4). Over the 9-year period, the counties with the highest mean PTB notification rates were the Hengdong, Sangzhi, Cili, Hengshan, and Fenghuang Counties. The global spatial autocorrelation analysis results showed that Moran’s I of the annual PTB notification rate at a county level ranged from 0.117 to 0.317 between 2014 and 2022 (*p* < 0.05) (Table 2), indicating that the occurrence of PTB exhibited a positive global spatial autocorrelation and was clustered.

**Table 2:** Global spatial autocorrelation analysis of the annual PTB notification rate in the Hunan Province, China from 2014 to 2022.

Year	Moran’s I	Z-score	*p* value	Pattern
2014	0.317	5.394	<0.001	Cluster
2015	0.239	4.111	<0.001	Cluster
2016	0.277	4.731	<0.001	Cluster
2017	0.117	2.080	0.037	Cluster
2018	0.222	3.845	<0.001	Cluster
2019	0.292	5.020	<0.001	Cluster
2020	0.284	4.874	<0.001	Cluster
2021	0.250	4.286	<0.001	Cluster
2022	0.230	3.964	<0.001	Cluster

PTB, pulmonary tuberculosis.

**Figure 5:**
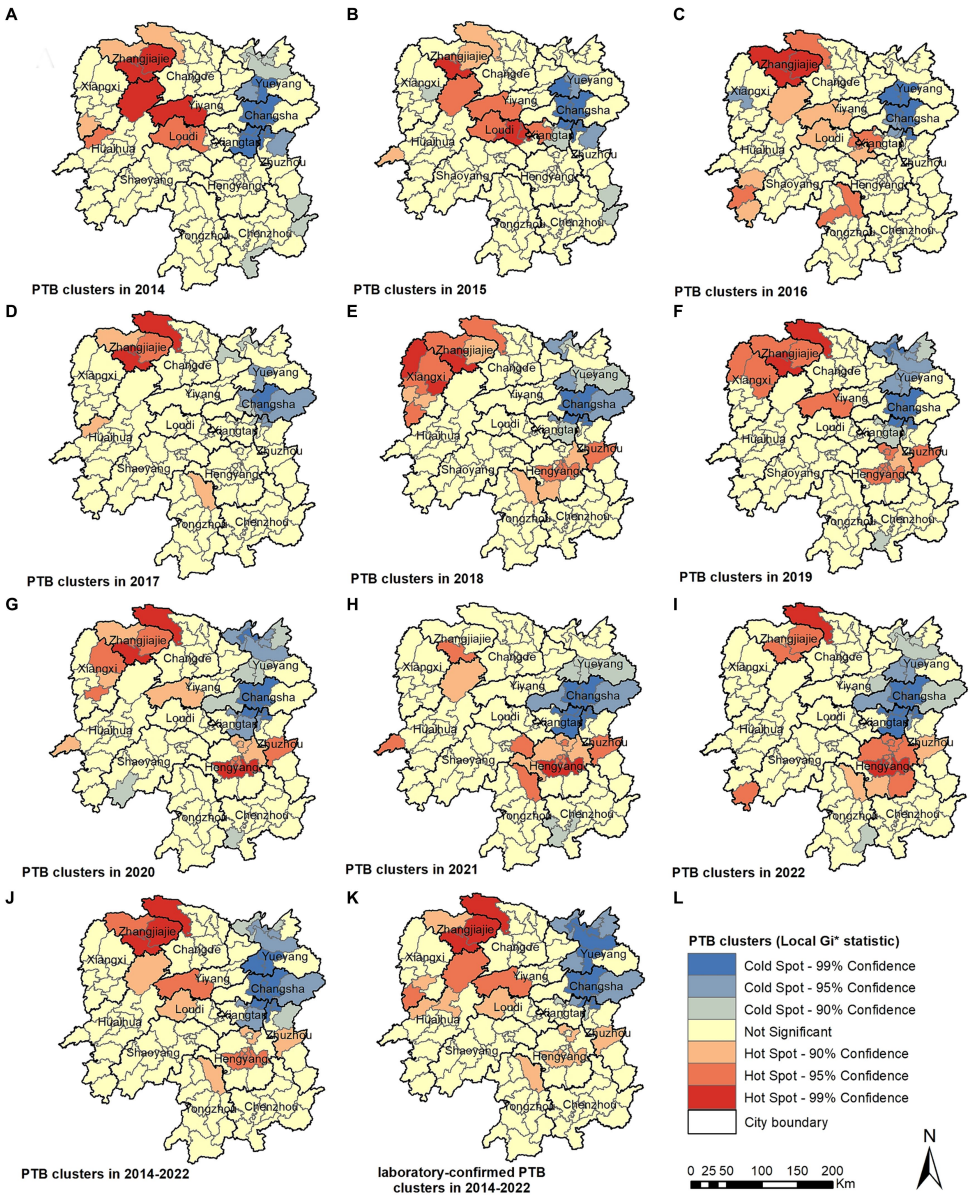
Pulmonary tuberculosis clusters in 2014 **(A)**, 2015 **(B)**, 2016 **(C)**, 2017 **(D)**, 2018 **(E)**, 2018 **(F)**, 2020 **(G)**, 2021 **(H)**, 2022 **(I)**, 2014–2022 **(J)**, laboratory-confirmed PTB clusters in 2014–2022 **(K)**.

The local spatial autocorrelation analysis results revealed that the hotspot and cold-spot areas of disease incidence in the Hunan Province changed annually from 2014 to 2022. The hotspot areas were mainly concentrated in the Xiangxi Autonomous Prefecture, Zhangjiajie City, and Hengyang City, and in some adjacent areas such as the Yuanling County in Huaihua City, Shimen County in Changde City, Xinhua County in Loudi City, Anhua County in Yiyang City, Qiyang City in Yongzhou City, and You County in Zhuzhou City. From 2018 to 2022, the hotspot areas in the Xiangxi Autonomous Prefecture gradually decreased annually and disappeared in 2021 and 2022; however, hotspot areas gradually formed in the Hengyang City from 2018 to 2022. The cold-spot areas of disease incidence gradually expanded from the urban zones of Changsha, Zhuzhou, and Xiangtan in 2014 to the entire cities of Changsha, Zhuzhou, and Xiangtan, and some areas of Yueyang, by 2022 (Figures 5A–I). Comparison of the mean PTB notification rate and the clustering of laboratory-confirmed cases of PTB over the 9-year study period revealed patterns in hotspot and cold-spot areas. However, the hotspot areas of laboratory-confirmed cases of PTB also included the Jishou City and Fenghuang County in the Xiangxi Autonomous Prefecture, and the Mayang Miao Autonomous County and Chenxi County in Huaihua City (Figures 5J,K).

### 3.4 Spatiotemporal clustering analysis using SaTScan

**Figure 6:**
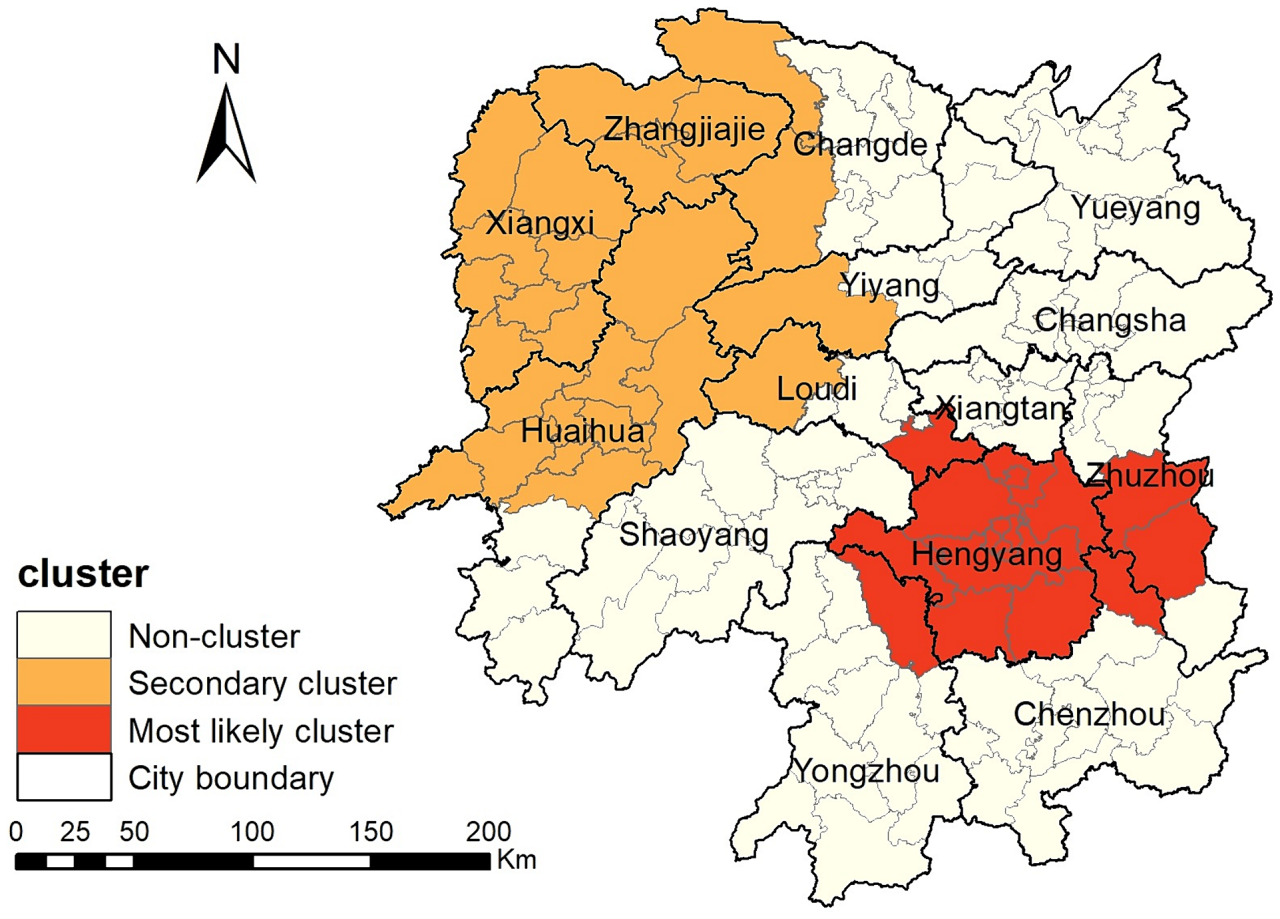
Space-time clusters of cases of pulmonary tuberculosis at a county level in the Hunan Province from 2014 to 2022.

The spatiotemporal scanning analysis results showed two distinct clusters of PTB incidence in the Hunan Province between 2014 and 2022. The most likely cluster was in the central-southern part of the Hunan Province, including the entire area of the Hengyang City, Anren County in Chenzhou City, Chaling County and You County in Zhuzhou City, Shuangfeng County in Loudi City, and Qiyang City in Yongzhou City, comprising 17 counties and districts. The clustering period was from March 1, 2018, to August 31, 2022, during which 47,338 cases of PTB were confirmed. The PTB notification rate within the clustered area was 1.36 times higher than that in areas outside the cluster. The secondary cluster was situated in the northwest region of the Hunan Province, encompassing the entire Xiangxi Autonomous Prefecture, Zhangjiajie City, eight county-level administrative regions in Huaihua City, Taoyuan County and Shimen County in Changde City, Anhua County in Yiyang City, and Xinhua County in Loudi City, comprising 25 counties and districts. The clustering period was from January 1, 2014, to May 31, 2018, during which 50,877 cases of PTB were notified. The PTB notification rate within the clustered area was 1.3 times higher than that in areas outside the cluster (Table 3; Figure 6).

**Table 3:** Space-time clustering of cases of PTB in the Hunan Province, China from January 2014 to December 2022.

Cluster type	Number of clustering areas	Cluster districts and counties	Cluster center/radius	Time frame	Observed cases	Expected cases	Relative risk	LLR	*p* value
Most likely cluster	17	Yanfeng District, Zhuhui District, Shigu District, Zhengxiang District, Hengnan County, Hengyang County, Hengdong County, Nanyue District, Hengshan County, Qidong County, Leiyang City, Changning City, Anren County, You County, Shuangfeng County, Qiyang City, Chaling County	(26.90 N, 112.62 E)/90.90 km	March 1, 2018, to August 31, 2022	47,338	35,785	1.36	1845.617	<0.001
Secondary cluster 1	25	Baojing County, Huayuan County, Guzhang County, Jishou County, Yongshun County, Yuanling County, Luxi County, Fenghuang County, Longshan County, Sangzhi County, Yongdi District, Chenxi County, Mayang County, Wulingyuan District, Xupu County, Hecheng District, Zhijiang County, Zhongfang County, Xinhuang County, Anhua County, Cili County, Hongjiang City, Taoyuan County, Shimen County, Xinhua County	(28.72 N, 109.65E)/194.20 km	January 1, 2014, to May 31, 2018	50,877	40,254	1.30	1423.918	<0.001

LLR, log-likelihood ratio; PTB, pulmonary tuberculosis.

## 4 Discussion

This study found that the PTB notification rate decreased from 2014 to 2022 in the Hunan Province. PTB case notifications exhibited spatial clustering, with clustering occurring predominantly in the central-southern and northwestern regions of the province. The PTB notification rate in the Hunan Province decreased from 85.88/100,000 in 2014 to 66.40/100,000 in 2022, showing a slow downward trend with fluctuations. This declining trend mirrors observations made in various provinces and cities across China in a national study (26), including the Chongqing Municipality (13), Shandong Province (27), and Hubei Province (28). The decrease in the tuberculosis epidemic is mainly attributable to the efforts made by the national and local governments in tuberculosis control. The Hunan Province has implemented multiple measures to enhance its capacity for tuberculosis control across the province, establishing a novel tuberculosis control system. It actively conducts health education to raise awareness among the population about tuberculosis prevention and treatment. Molecular biology testing is widely promoted in prefectures and counties to achieve rapid diagnosis of PTB, leading to a gradual increase in the proportion of patients with microbiological confirmation and further improvement in the quality of tuberculosis diagnosis. Active screening is performed among key populations such as close contacts of patients with laboratory-confirmed PTB, older adults, and patients with diabetes patients to diagnose cases early, promptly initiate treatment, and reduce the spread of tuberculosis. In educational institutions, tuberculosis management has been strengthened by implementing PTB screening for students before entry into primary schools, middle schools, and high schools, as well as universities, controlling the entry of students with a high risk of developing tuberculosis. Additionally, preventive treatment for latent tuberculosis infection with a high risk of disease progression is promoted, reducing the occurrence of tuberculosis outbreaks in schools.

Our study found that the monthly PTB notifications showed significant seasonal variation, with peaks in January or March and troughs in December. The Hunan Province is located between 25° and 30° north latitude and has a subtropical monsoon climate. January is typically a winter month and is characterized by damp and rainy weather with little sunlight. Lack of exposure to ultraviolet light and poor ventilation are known risk factors for tuberculosis transmission (29). February coincides with the Chinese Lunar New Year holiday, when traditional customs discourage seeking medical care, leading to a higher PTB notification rate in March after the holiday period. This cyclical pattern is similar to research findings in the Chongqing Municipality (13), Hubei Province (28), Guangxi Province (30), and Hunan Province (15) but differs from the results observed in the Zhejiang Province (11), Taiwan (31) and Hong Kong (32). However, this pattern was disrupted from 2020 to 2022, with the peak in notifications delayed to June, April, and July in 2020, 2021, and 2022, respectively, although the trough still occurred in December each year. This may be attributed to the COVID-19 pandemic, which led to successive enforcement of control measures by the national and local governments based on the prevalence of COVID-19. People’s movements were affected, leading to delays in accessing healthcare services and diagnosis in individuals with PTB, thus disrupting the seasonal pattern of PTB notifications (33, 34).

This study identified significant spatial clustering of PTB notifications in the Hunan Province, with hotspot areas primarily concentrated in the Xiangxi Autonomous Prefecture, Zhangjiajie City, and Hengyang City, similar to the findings of a previous study carried out in the Hunan Province in 2012 and 2013 (16). The hotspot areas in the Xiangxi Autonomous Prefecture gradually decreased over the years, disappearing by 2021 and 2022, whereas hotspot areas gradually formed in the Hengyang City from 2018 to 2022. Space-time scan analysis revealed that the most likely clustering area was around the Hengyang City, encompassing 17 adjacent counties and districts, with clustering occurring from March 1, 2018, to August 31, 2022. A secondary clustering area was identified around the Xiangxi Autonomous Prefecture and Zhangjiajie City, covering 25 adjacent counties and districts, with clustering occurring from January 1, 2014, to May 31, 2018. Local spatial autocorrelation analysis of hotspot areas was consistent with the clustering areas identified by space-time scan analysis, confirming the stability and reliability of the research results. The Xiangxi Autonomous Prefecture and Zhangjiajie City, located in the northwestern region of the province, are economically underdeveloped, with poor living conditions for residents and a lack of medical and health resources. Patients in these areas often come from impoverished families and may not receive timely diagnosis and treatment, causing the transmission of PTB and contributing to a higher incidence of the disease (35, 36). In contrast, the Xiangxi Prefecture is an area in which ethnic minorities (Tujia and Miao) are concentrated. People belonging to ethnic minorities have an increased risk of delayed diagnosis of tuberculosis (37). In 2021, the PTB notification hotspot in the Zhangjiajie City decreased significantly, which may have been related to the COVID-19 epidemic in the Zhangjiajie City in July and August 2021 and the implementation of epidemic control measures. The COVID-19 pandemic resulted in reduced healthcare-seeking behavior among individuals with tuberculosis, affecting the diagnosis and treatment (34, 38). This study also identified a cold-spot area that gradually expanded from the urban zones of Changsha, Zhuzhou, and Xiangtan in 2014 to encompass the entire city of Changsha, the urban zones of Zhuzhou and Xiangtan, and parts of Yueyang. This finding is generally similar to that of a previous study (15). These areas are the most economically developed region in the Hunan Province, with abundant medical resources and strong public awareness of disease prevention and treatment.

To our knowledge, this is the first space-time scan analysis of spatiotemporal clustering of PTB notifications at a county level in the Hunan Province. It identified regions with high PTB notification rates in the Hunan Province, providing valuable insights for formulating more effective control strategies.

This study has few limitations. First, the data were sourced from the National Disease Surveillance System, which may have resulted in an underestimation of the PTB epidemic in the Hunan Province due to some cases not being notified. Second, due to the adjustment of the national tuberculosis diagnostic criteria, cases of tuberculous pleurisy have been included in the pulmonary tuberculosis reporting system since May 1, 2018, which may introduce a slight bias in the research results. Third, this study used the space-time scan method to investigate PTB clustering. This method depends on circular spatial scan windows and space-time cylinders, which may not work well in irregular spaces, potentially leading to instability in determining clusters. Finally, this research examined only the spatiotemporal distribution patterns of tuberculosis without investigating individual factors and socioeconomic factors that may influence the incidence of tuberculosis. Some studies have found that personal factors such as smoking (39), alcohol consumption (40, 41), HIV (42), and diabetes (43), as well as social factors such as poverty (36), population mobility (44, 45), healthcare resources (46), and environmental pollution (47, 48) may influence the distribution of tuberculosis. Future studies should aim to restrict the geographical unit of analysis to the township level and explore how these factors affect the incidence of PTB.

## 5 Conclusion

This study characterized the temporal trend and spatial distribution of tuberculosis in the Hunan Province. PTB diagnosis peaked in the spring. The PTB notification rate in the Hunan Province exhibited spatial clustering, concentrated mainly in the central-southern and northwestern regions of the province. This suggests that priority should be given to strengthening tuberculosis control efforts in these regions.

## Data availability statement

The datasets presented in this article are not readily available because they are sourced from the Infectious Disease Information System and all data remain confidential. Requests to access the datasets should be directed to GH, 18774994296@163.com.

## Ethics statement

The studies involving humans were approved by Medical Ethics Committee of Hunan Chest Hospital. The studies were conducted in accordance with the local legislation and institutional requirements. Written informed consent for participation was not required from the participants or the participants’ legal guardians/next of kin in accordance with the national legislation and institutional requirements.

## Author contributions

GH: Data curation, Funding acquisition, Methodology, Software, Visualization, Writing – original draft. ZX: Data curation, Writing – review & editing. LB: Supervision, Writing – review & editing. JL: Conceptualization, Supervision, Writing – review & editing. SY: Software, Supervision, Writing – review & editing. HY: Methodology, Supervision, Writing – review & editing.
